# Bio-Inspired Central Pattern Generator for Adaptive Gait Generation and Stability in Humanoid Robots on Sloped Surfaces

**DOI:** 10.3390/biomimetics10090637

**Published:** 2025-09-22

**Authors:** Junwei Fang, Yinglian Jin, Binrui Wang, Kun Zhou, Mingrui Wang, Ziqi Liu

**Affiliations:** 1 College of Metrology Measurement and Instrument, China Jiliang University, Hangzhou 310018, China; fangjwpaper@163.com; 2 The State Key Laboratory of Robotics, Shenyang Institute of Automation, Shenyang 110016, China; 19a0102168@cjlu.edu.cn; 3 College of Mechanical and Electrical Engineering, China Jiliang University, Hangzhou 310018, China; jinyinglian@cjlu.edu.cn (Y.J.); 18867135831@163.com (M.W.); zqliu@cjlu.edu.cn (Z.L.); 4 The Zhejiang Province Key Laboratory of On-Line Testing Equipment Calibration Technology Research, China Jiliang University, Hangzhou 310018, China

**Keywords:** humanoid robot, central pattern generator, constrained evolutionary optimization, adaptive walking, reflex regulation

## Abstract

Existing research has preliminarily achieved stable walking in humanoid robots; however, natural human-like leg motion and adaptive capabilities in dynamic environments remain unattained. This paper proposes a bionic central pattern generator (CPG) gait generation method based on Kimura neurons. The method maps the CPG output to the spatial motion patterns of the robot’s center of mass (CoM) and foot trajectory, modulated by 22 undetermined parameters. To address the vague physical interpretation of CPG parameters, the strong neuronal coupling, and the difficulty of decoupling, this research systematically optimized the CPG parameters by defining an objective function that integrates dynamic balance performance with step constraints, thereby enhancing the naturalness and coordination of gait generation. To further enhance the walking stability of the robot under varying road curvatures, a vestibular reflex mechanism was designed based on the Tegotae theory, enabling real-time posture adjustment during slope walking. To validate the proposed approach, a virtual simulation platform and a physical humanoid robot system were constructed to comparatively evaluate motion performance on flat terrain and slopes with different gradients. The results show that the energy consumption characteristics of robot-coordinated gait are highly consistent with the energy-saving mechanism of human natural motion. In addition, the established reflection mechanism significantly improves the motion stability of the robot in slope transition, and its excellent stability margin and environmental adaptability are verified by simulation and experiment.

## 1. Introduction

Humanoid robots, with their bionic design and highly flexible structures, have the potential to mimic human behaviors in complex environments. However, the strong coupling among multiple degrees of freedom presents significant challenges in motion control [1,2]. While prior studies have achieved stable robot locomotion in structured environments [3,4], critical issues such as maintaining stability during terrain transitions and ensuring coordinated gait movements remain unresolved.

In the study of humanoid robot motion stability, the integration of model predictive control (MPC) and whole-body control (WBC) forms a well-established framework for bipedal walking control [5,6]. However, this control approach relies on a dynamic model to generate gait trajectories that satisfy dynamic balance constraints. During environmental interactions, it requires solving high-dimensional optimization problems in real time. Consequently, its response speed and generalization capability are constrained by model inaccuracies and computational latency, rendering it unsuitable for unstructured environments or unexpected disturbances [7,8]. As a biologically-inspired control paradigm, the Central Pattern Generator (CPG) offers a biomimetic approach for humanoid robot motion control by emulating the neural mechanisms underlying rhythmic movement regulation in vertebrates [9,10,11].

The CPG typically comprises a network of coupled nonlinear oscillators that generate phase-locked rhythmic signals through neuronal excitatory connections and lateral inhibition mechanisms [12,13]. Through dynamic phase coupling, the CPG enables real-time adjustment of inter-neuronal coordination. This capability facilitates natural synchronization of humanoid robots’ lower-limb kinematic chains while preventing the unnatural motion artifacts characteristic of traditional discretized gait-generation methods [14,15]. The CPG typically produces sinusoidal-like periodic output signals whose characteristics are precisely governed by model parameters. When encountering complex terrains or unexpected disturbances, minimal parameter adjustments to the CPG network can elicit stable locomotion patterns. This approach offers significant advantages in computational efficiency and environmental adaptability compared to traditional control methods [16,17]. However, the parameter space of CPG is typically characterized by high dimensionality, and parameter tuning is frequently guided by empirical heuristics due to the complex nonlinear relationships involved. Achieving optimal parameter configurations that simultaneously satisfy periodic motion requirements, walking stability, and inter-limb coordination presents a significant control challenge. Therefore, the optimization of CPG network parameters has become one of the key directions of current research. Ruppert and Spröwitz [18] developed a bio-inspired CPG control framework for quadruped robots that employs Bayesian optimization for dynamic parameter adaptation, specifically targeting energy efficiency optimization. Wang et al. [19] designed a multi-layer CPG network to generate the swimming rhythm of robotic fish, and optimized the CPG parameters using the PSO algorithm with the average swimming speed as the goal. Zhang et al. [20] proposed an enhanced PSO algorithm to optimize the CPG controller parameters for humanoid robot joints, with the specific objective of minimizing lateral deviation during straight-line walking locomotion. Wu et al. [21] employed multivariate linear mapping to transform CPG parameter distribution analysis into a coefficient adjustment problem, enabling stable humanoid locomotion. Wang et al. [22] combined offline genetic algorithm optimization of CPG rhythm parameters with online reinforcement learning of a high-level neural network for dynamic parameter adjustment, enabling multimodal motion control. Current research predominantly employs reinforcement learning or multi-objective optimization algorithms for dynamic CPG parameter optimization, with the primary objective of balancing competing constraints among rhythmic stability, energy efficiency, and environmental adaptability. However, the anthropomorphic naturalness of humanoid locomotion is frequently neglected as a critical performance metric. Developing computationally efficient parameter optimization strategies that simultaneously enhance gait naturalism and maintain dynamic stability remains a fundamental challenge in advancing CPG controller performance.

Based on the biomimetic principles of biological neural circuits, CPG network models can be categorized into two distinct forms: non-feedback and feedback [23,24]. Among these, feedback-enabled CPG networks can directly incorporate environmental information and dynamically modulate rhythmic signals through biologically-inspired reaction mechanisms, thereby significantly enhancing robotic adaptability and robustness in complex environments [25,26]. Tran et al. [27] developed an adaptive CPG network architecture that integrates sensory neurons with motor neurons through synaptic coupling, demonstrating robust state recovery from external perturbations via feedback-enabled CPG mechanisms. Song et al. [28] emulated the neural signal transmission characteristics of biological CPG systems by implementing a delayed coupling feedback mechanism, enabling the flexible generation and seamless transition of multimodal gaits in quadruped robots. Owaki et al. [29] introduced the Tegotae concept, establishing a computationally tractable adaptive criterion for bio-inspired CPG control. This framework quantitatively evaluates the congruence between proprioceptive feedback and locomotor intent, enabling autonomous gait adaptation. Wang et al. [30] employed the Tegotae principle to quantify muscle contraction intent and developed a bio-inspired reflex control system capable of mitigating joint impact disturbances. Herneth et al. [31] leveraged the Tegotae framework to quantify the congruence between CPG-generated control signals and contact forces, achieving global motion adaptation using only single-point force feedback. The environmental adaptability and disturbance robustness of the system are substantially enhanced through integration of feedback signals into the control method, enabling real-time modulation of dynamic properties in the CPG network.

This study presents a bio-inspired hierarchical control framework that synergistically combines CPG-based rhythm generation with Tegotae feedback regulation. By incorporating gait coordination evaluation metrics, a multi-objective optimization strategy is developed to address the challenges of dynamic stability and motion coordination in humanoid robots navigating terrains with abrupt curvature variations. The key contributions of this work are outlined as follows:

(1) Based on an analysis of human walking gait characteristics, a spatial trajectory planner for humanoid robots was designed using the Kimura CPG model. This planner generates CoM and foot-end trajectories that mimic human gait patterns.

(2) To address the issues of environmental adaptability and stability in humanoid robot locomotion, a multi-objective optimization scheme for CPG parameters was developed, coupling ZMP stability constraints with gait coordination conditions.

(3) A perception-feedback matching function was constructed using the Tegotae theory to simulate the human vestibular reflex mechanism, enabling rapid postural adjustments during walking on sloped surfaces.

**Figure 1:**
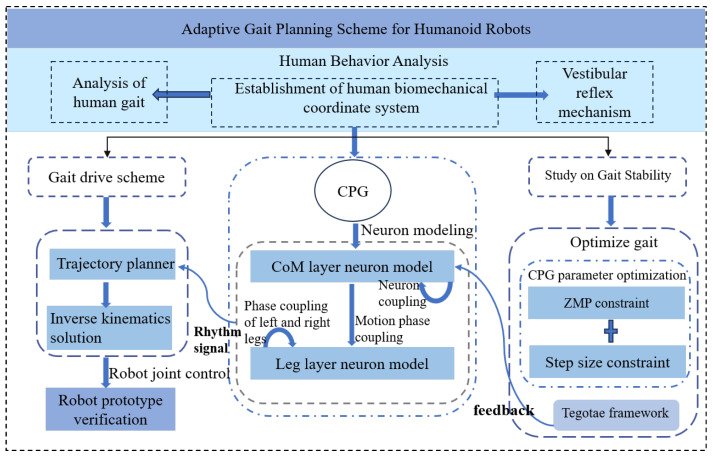
CPG bionic gait research framework.

The structure of this paper is organized as follows: Section 1 is the introduction section, which describes the research status and trends of CPG in walking control of humanoid robots. Section 2 explores the development of the Kimura CPG neuron model based on the movement characteristics of the human body and details the design of specific spatial motion planning models. Section 3 delves into the theoretical foundation for achieving stable walking in humanoid robots and elaborates on the rules for CPG parameter modulation. Section 4 presents simulations and physical experiments of humanoid robot walking to validate the effectiveness of the proposed gait planning method, which integrates an optimization algorithm with CPG. The research concepts presented in this paper and the framework for CPG-based bionic gait design are illustrated in Figure 1.

## 2. Motion Trajectory Generation Based on CPG

### 2.1. Description of Human Motion Model

**Figure 2:**
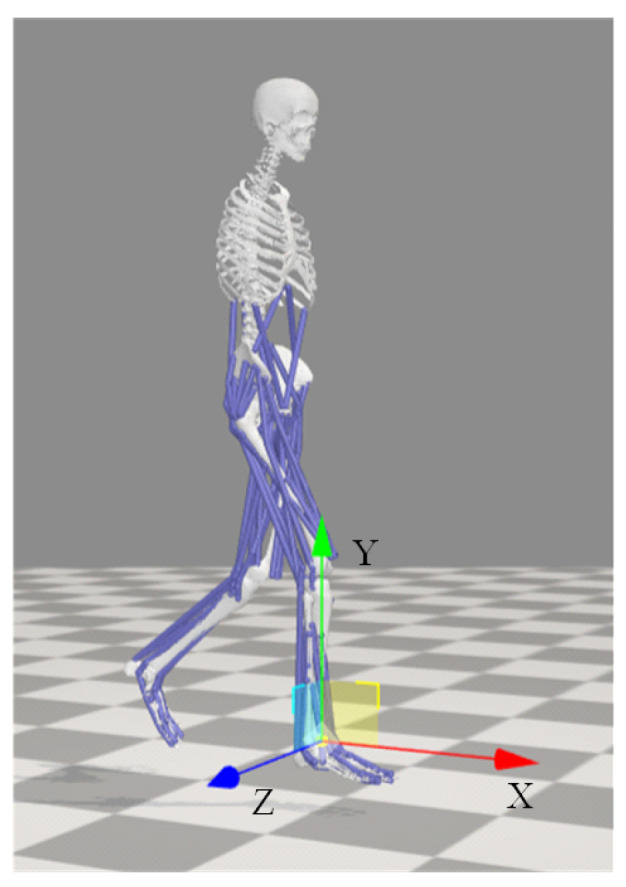
The positive *x*-axis defines the forward direction, the *y*-axis aligns with the direction of gravity, and the *x*-*z* plane constitutes the horizontal plane, with the *x*-, *y*-, and *z*-axes represented by the red, green, and blue components, respectively.

In this study, a global coordinate system for human motion analysis was established based on the biomechanical coordinate system definition standards of the International Society of Biomechanics (ISB) [32]. As shown in Figure 2, the *y*-axis represents the direction parallel to gravity, while the *x*-axis and *z*-axis lie within the sagittal plane and perpendicular to the *y*-axis, pointing forward and to the right, respectively.

**Figure 3:**
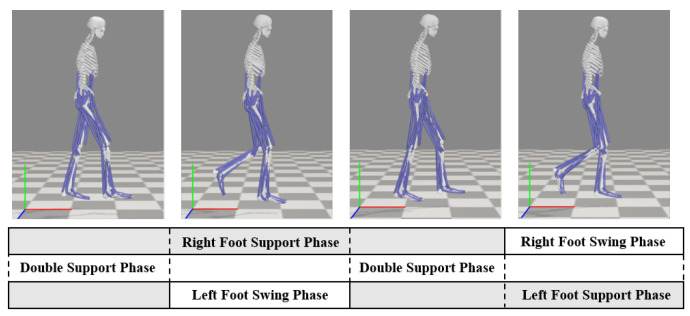
Human walking phase diagram.

Human lower-limb walking is a smooth and stable motion achieved under the coordination and control of multiple joints by the nervous system, with movement synchronization governed by the CPG network. Walking simulations were conducted in OpenSim using an open-source human musculoskeletal model, and the gait characteristics during human walking were analyzed. A schematic diagram of the gait phases within one walking cycle is shown in Figure 3. For a single leg, the swing phase accounts for approximately 40% of the gait cycle, while the stance phase accounts for around 60%.

In the global coordinate system, foot trajectories are decomposed into x/y/z components for analysis. The *x*-directional trajectory of both feet demonstrates a periodically increasing trend with time-varying slope characteristics. For individual feet, an approximate phase difference of π/2 is observed between *x* and *y* directional motions, while between contralateral feet in the same direction, the phase difference measures approximately π.

### 2.2. Concept and Design of the CPG Model

**Figure 4:**
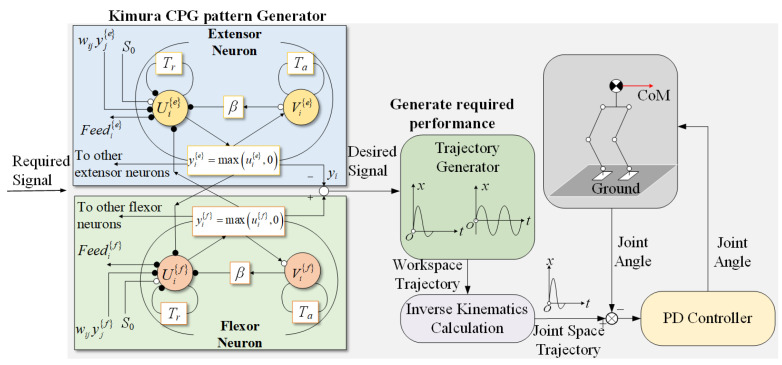
Kimura neuron structure and CPG control strategy for humanoid robots.

The biological CPG is a low-level neural center that autonomously produces periodic rhythmic signals through self-stimulation. The Kimura oscillator is a neural oscillator model based on mutually inhibitory neuron pairs. In this paper, Kimura neuron oscillators were employed to simulate the behavior of biological CPG for robotic control. This oscillator generates rhythm signals by coupling two units representing flexor and extensor motor neurons, and its combined output is the control signal that drives joint movement. The mathematical representation of a pair of antagonistic neurons is provided in Equation (1).

(1) $$\begin{array}{c} T_{r}{\dot{u}}_{i}^{e}=-u_{i}^{e}+\alpha y_{i}^{f}-\beta v_{i}^{e}+\sum_{j=1}^{n}w_{ij}y_{j}^{e}+feed_{i}^{e}+s_{i}^{e}\\ T_{a}{\dot{\nu }}_{i}^{e}=-\nu_{i}^{e}+y_{i}^{e}\\ T_{r}{\dot{u}}_{i}^{f}=-u_{i}^{f}+\alpha y_{i}^{e}-\beta v_{i}^{f}+\sum_{j=1}^{n}w_{ij}y_{j}^{f}+feed_{i}^{f}+s_{i}^{f}\\ T_{a}{\dot{\nu }}_{i}^{f}=-\nu_{i}^{f}+y_{i}^{f}\\ y_{i}^{\left\{e,f\right\}}=\max\left(u_{i}^{\left\{e,f\right\}},0\right)\\ y_{hi}=-u_{i}^{e}+u_{i}^{f}\\ y_{ki}=\left\{\begin{array}{cc} v_{i}^{e}-v_{i}^{f}, & v_{i}^{e}-v_{i}^{f}>0\\ 0, & v_{i}^{e}-v_{i}^{f}<0 \end{array}\right. \end{array}$$

where *i*, *f*, and *e* represent the *i*-th oscillator, flexor, and extensor neurons, respectively; $T_{r}$ and $T_{a}$ denote the rise time constant and adaptation time constant, which influence the output period of the oscillator; $u_{i}^{\left\{e,f\right\}}$ and $v_{i}^{\left\{e,f\right\}}$ represent the internal and inhibitory states of the extensor and flexor neurons, respectively; $w_{ij}$ represents the connection weights between neurons, affecting the output phase of the oscillator; $s_{i}$ is the external excitation input, while $feed_{i}$ represents the external feedback input, both of which influence the output amplitude; $w_{fe}$ is the mutual inhibition coefficient between cells, and β is the adaptation coefficient; both $y_{hi}$ and $y_{ki}$ are available as outputs for the *i*-th oscillator, in this work, we select $y_{hi}$ as the output. The corresponding structure of neural units and the CPG control strategy for humanoid robots are shown in Figure 4.

To control the robot to simulate human walking, the CPG oscillators need to output rhythmic signals that conform to the walking pattern of the human body. Currently, one of the commonly adopted approaches involves utilizing CPG as motion planners in the joint space of robots. However, due to the high degrees of freedom in humanoid robots, implementing such methods requires the integration of extensive CPG networks for control, which significantly complicates parameter tuning. The centroid and foot CPG trajectory generation method demonstrates superior computational efficiency and motion stability compared to joint space planning approaches, achieving robust performance with fewer neural oscillators [33,34].

**Figure 5:**
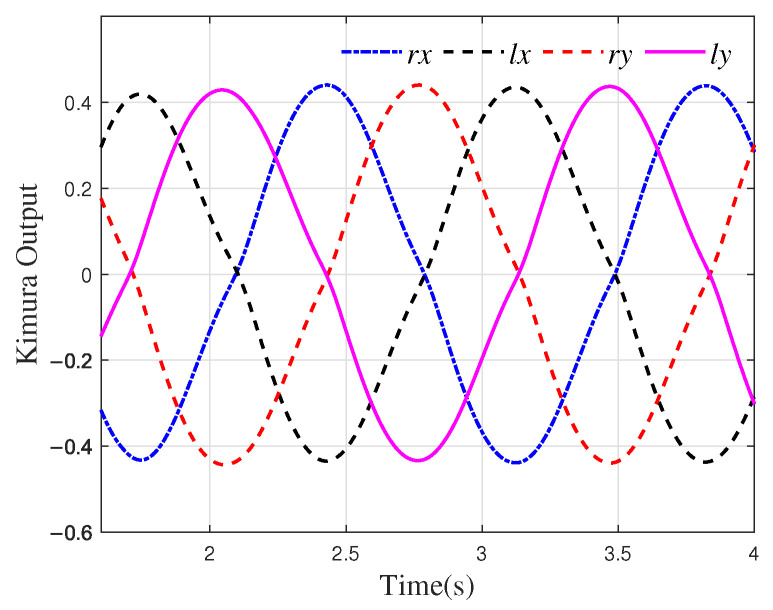
Kimura oscillator output.

Based on the simulation results obtained from OpenSim, the outputs of the CPG designed to generate the motion components in the *x* and *y* directions for the left and right feet of the robot are denoted as $l_{x}$, $l_{y}$, $r_{x}$, and $r_{y}$. Here, *l* and *r* correspond to the left and right feet, respectively, while the subscripts indicate the *x* and *y* components. The neuron connections are configured as inhibitory connections, i.e., $w_{ij}=-1\;\left(i\neq j\right)$. The parameters are set as $T_{r}=0.2$, $T_{a}=0.4$, $s_{0}=0.6$, β=3.5, a=−2.0. The output of the oscillators with these settings is shown in Figure 5.

The connection weights $w_{ij}$ determine the phase relationship of the output of the oscillator. Here, $l_{x}\left(r_{x}\right)$ and $l_{y}\left(r_{y}\right)$ maintain a phase difference of π/2, while the phase difference between $l_{x}\left(l_{y}\right)$ and $r_{x}{(}_{y})$ is π. Additionally, during walking, the CoM of the robot should adjust within the *x*-*z* plane in relation to the foot and body posture angles. When the robot is in single-leg support, the CoM needs to shift in the *z* direction toward the foot contact point to prevent tipping. Therefore, the CPG signal for the movement of the CoM in the *z* direction should remain in phase with one of the feet, ensuring stability during single-leg support. By solving inverse kinematics, the spatial trajectories of the CoM and foot end can be converted into joint space for control [35].

### 2.3. CPG-Based Trajectory Generators

Human walking shows linearly increasing *x*-direction displacement of foot and CoM with periodic oscillations, whereas standard CPG outputs lack this linear amplitude variation. Moreover, it is difficult to achieve precise waveform modulation through parameter adjustment alone. In this study, the output signal of the CPG is mapped to the motion trajectory of the foot-end. The mapping function for the left foot walking trajectory in the sagittal plane is designed as shown in Equation (2).

(2) $$\begin{array}{c} foot_{x}^{l}=x_{0}+C_{x}l_{x}+\delta_{f}t\\ foot_{y}^{l}=y_{0}+C_{y}l_{y} \end{array}$$

where $x_{0}$ and $y_{0}$ represent output offsets, used to adjust the initial position of the center of gravity along the *x*-axis and *y*-axis; $C_{x}$ and $C_{y}$ are gain coefficients controlling the amplitude of the rhythmic signal to adjust stride length variation; and $\delta_{f}t$ introduces a linear time-varying factor, simulating the effect of the gait in the *x* direction, in which the motion component increases linearly with time. The outputs of the oscillators are represented by $l_{x}$ and $l_{y}$. The mapping function for the trajectory of the right foot in the sagittal plane is shown in Equation (3).

(3) $$\begin{array}{c} foot_{x}^{r}=x_{0}+C_{x}r_{x}+\delta_{f}t\\ foot_{y}^{r}=y_{0}+C_{y}r_{y} \end{array}$$

where $r_{x}$ and $r_{y}$ are the outputs of the oscillators, so the phase difference between $foot_{x}^{r}$ and $foot_{y}^{r}$ is denoted as π/2, and the phase difference between $foot_{x}^{l}\left(foot_{y}^{l}\right)$ and $foot_{x}^{r}\left(foot_{y}^{r}\right)$ is denoted as π.

Let the outputs of the CoM CPG output be $\vartheta_{x}$, $\vartheta_{y}$, and $\vartheta_{z}$. The corresponding mapping function in Cartesian space is presented in Equation (4).

(4) $$\begin{array}{c} {\mathrm{CoM}}_{x}=\eta_{x}+\varsigma_{x}\vartheta_{x}+\lambda_{c}t\\ {\mathrm{CoM}}_{y}=\eta_{y}+\varsigma_{y}\vartheta_{y}\\ {\mathrm{CoM}}_{z}=\eta_{z}+\varsigma_{z}\vartheta_{z} \end{array}$$

where ${\mathrm{CoM}}_{x}$, ${\mathrm{CoM}}_{y}$, and ${\mathrm{CoM}}_{z}$ represent the components of the CoM trajectory in the *x*, *y*, and *z* directions, respectively; $\eta_{x}$, $\eta_{y}$, and $\eta_{z}$ represent the initial offsets in the *x*, *y*, and *z* directions; $\varsigma_{x}$, $\varsigma_{y}$ and $\varsigma_{z}$ are gain coefficients; and $\lambda_{c}t$ is the time-related term.

The newly generated motion signals not only preserve the periodic rhythmic characteristics and phase relationships of the original CPG but also modulate their positional amplitude through the application of initial offsets and gain coefficients. Additionally, the introduction of time-related terms incorporates linear dynamic changes. This transformation allows the robot control system to more precisely control the position of the CoM and lower limbs, enabling better adaptation to different surface conditions and control objectives.

## 3. Research on Stable Walking and Environmental Adaptability

### 3.1. Exploring Walking Stability with ZMP Criterion

**Figure 6:**
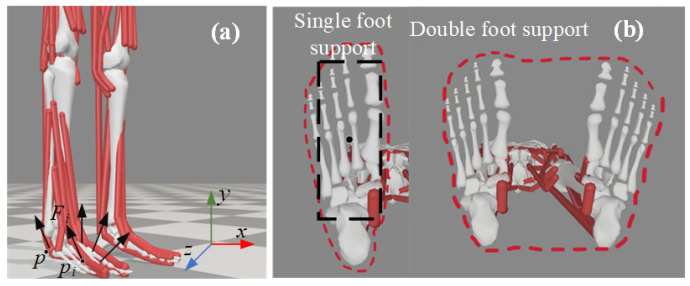
ZMP Element Diagram. (**a**) Schematic diagram of foot and ground torque. (**b**) ZMP stability region diagram, single foot support, and bipedal support. The red dashed line indicates the stable envelope region, while the black rectangle denotes the nominal contact area of the sole used in this study.

Through the design of the foot-end and CoM trajectory generators, a set of three-dimensional trajectories can be generated that shape-wise resemble human gait characteristics. However, these generated trajectories are insufficient to ensure the stable walking of the humanoid robot in space. For a moving humanoid robot, a practical criterion for gait stability is the ZMP stability criterion. The ZMP is the point at which the reaction moment from the ground on the foot is zero. When the ZMP lies within the contact area between the foot and the ground during the motion of the robot, the robot is considered to be stable and unlikely to tip over, and the gait is deemed stable. To illustrate the derivation of the ZMP, the force vectors acting on the foot are shown schematically in Figure 6a. Let the coordinates of the center of gravity be $p_{c}=\left[p_{cx},p_{cz},p_{cy}\right]$, and the coordinates of the ZMP be $p={\left[p_{x},p_{z},p_{y}\right]}^{T}$. The ground exerts a distribution of reaction forces at various contact points. The ZMP can be described by Equation (5).

(5) $$p=\frac{\underset{i=1}{\overset{N}{\Sigma }}p_{i}F_{iy}}{\underset{i=1}{\overset{N}{\Sigma }}F_{iy}}=\underset{i=1}{\overset{N}{\Sigma }}\frac{F_{iy}}{\underset{i=1}{\overset{N}{\Sigma }}F_{iy}}p_{i}$$

where $p_{i}$ represents the discrete contact points between the foot and the ground, i=(1,2,…,N), $F_{i}={\left[F_{ix},F_{iz},F_{iy}\right]}^{T}$ denotes the forces exerted on the foot of the humanoid robot by the ground at each of these contact points. The ZMP torque $\tau_{p}$ is shown in Equation (6).

(6) $$\left\{\begin{array}{c} \tau_{px}=\sum_{i=1}^{N}\left(p_{iz}-p_{z}\right)F_{iy}-\sum_{i=1}^{N}\left(p_{iy}-p_{y}\right)F_{iz}\\ \tau_{pz}=\sum_{i=1}^{N}\left(p_{iy}-p_{y}\right)F_{ix}-\sum_{i=1}^{N}\left(p_{ix}-p_{x}\right)F_{iy}\\ \tau_{py}=\sum_{i=1}^{N}\left(p_{ix}-p_{x}\right)F_{iz}-\sum_{i=1}^{N}\left(p_{iz}-p_{z}\right)F_{ix} \end{array}\right.$$

Assuming the footplate is the reference plane, we design the relevant components as $\tau_{x}=\tau_{z}=0$ and $p_{y}=p_{iy}$. The torque relationship at the origin can be obtained as follows:

(7) $$\tau =\tau_{p}+\sum_{i=1}^{N}p\times F_{i}$$

The system momentum $P=\left[P_{x},P_{y},P_{z}\right]$ and angular momentum $L=\left[L_{x},L_{y},L_{z}\right]$ around the origin are shown in Equation (8) and Equation (9), respectively.

(8) $$\dot{P}=Mg+\sum_{i=1}^{N}F_{i}$$

(9) $$\dot{L}=p_{c}\times Mg+\tau$$

where *M* is the total mass of the system, and *g* represents the acceleration due to gravity. Substituting Equations (7) and (8) into Equation (9) yields:

(10) $$\tau_{p}=\dot{L}-p_{c}\times Mg+\left(\dot{P}-Mg\right)\times p$$

Simplify the robot into a pointing model, where the torque in the *x* and *z* directions is 0. The calculation formula for the ZMP point can be derived as follows:

(11) $$\begin{array}{c} p_{x}=p_{cx}-\frac{\left(p_{cy}-p_{y}\right){\ddot{p}}_{cx}}{{\ddot{p}}_{cy}+g}\\ p_{z}=p_{cz}-\frac{\left(p_{cy}-p_{y}\right){\ddot{p}}_{cz}}{{\ddot{p}}_{cy}+g} \end{array}$$

Another important concept in the ZMP stability criterion is the concept of the stability region. During the single-support phase, the stability region corresponds to the contact area of the single foot with the ground. During the double-support phase, the stability region is the smallest convex polygon that encloses the contact areas of both feet with the ground, as shown in Figure 6b. The region enclosed by the red dashed line represents the stability region. To simplify the calculation process, this paper removes the irregular portions around the contact area of the foot, retaining only a rectangular region with an area smaller than that of the foot.

Based on the above analysis, when the robot walks along a straight line, the ZMP stability region is denoted as *D*. The stability margin $D_{s}$ during the walking process of the robot increases as the gait becomes more stable. The calculation method for $D_{s}$ is shown in Equation (12).

(12) $$D_{s}=\min\left({\mathrm{ZMP}}_{x}-S_{x},{\mathrm{ZMP}}_{z}-S_{z}\right)$$

where ${\mathrm{ZMP}}_{x}$ and ${\mathrm{ZMP}}_{z}$ represent the coordinates of the ZMP on the plane, while $S_{x}$ and $S_{z}$ denote the boundary values of the contact area envelope in the *x*-direction and *z*-direction, respectively.

### 3.2. Parameter Optimization Through Genetic Algorithm Approach

Unlike other multi-legged robots that possess self-stabilizing characteristics in terms of their mechanism, humanoid robots need to simultaneously plan the motion trajectories of both the CoM and the feet in order to achieve stable walking. Compared to joint-based CPG control methods for robot motion, the CPG planner for the CoM and foot trajectory reduces the number of internal parameters. For the robot model in this research, the number of parameters has significantly decreased from 50 in the joint space method to 22. However, the coupling relationships between these parameters are more complex. Traditional empirical tuning methods are clearly insufficient for meeting application needs. In this paper, a genetic algorithm is employed to evolve and optimize both the internal parameters of the CPG and the external combination coefficients, enabling precise modulation of the CPG and foot trajectories.

The foot trajectory generator designed in this paper consists of four CPG neurons, while the CoM trajectory generator consists of three CPG neurons. With the CPG internal parameters and external combination coefficients as decision variables, each generation of the population contains 22 individuals. To ensure that the optimized CPG parameters via the genetic algorithm can stably produce rhythmic signals, constraint conditions are designed as shown in Equation (13).

(13) $$\left\{\begin{array}{c} \;\beta \geq w_{fe}-1\\ w_{fe}>1+T_{r}/T_{a} \end{array}\right.$$

Two fitness functions are formulated to achieve stable bipedal locomotion: $fitness_{\mathrm{ZMP}}$, based on ZMP stability criteria, and $fitness_{\mathrm{dev}}$, which quantifies lateral deviation during walking. $fitness_{\mathrm{ZMP}}$ is shown in Equation (14).

(14) $$fitness_{\mathrm{ZMP}}=1/\sqrt{{\left({\mathrm{ZMP}}_{x}-x_{\mathrm{cen}}\right)}^{2}+{\left({\mathrm{ZMP}}_{z}-z_{\mathrm{cen}}\right)}^{2}}$$

where ($x_{\mathrm{cen}}$, $z_{\mathrm{cen}}$) represents the center of the stability region in each gait cycle. $fitness_{\mathrm{ZMP}}$ is designed to find the values closest to the center coordinates of the stability region, ensuring that the ZMP falls within the stability region and that the value of $D_{s}$ is maximized. $fitness_{\mathrm{dev}}$ is shown in Equation (15).

(15) $$fitness_{\mathrm{dev}}=\frac{1}{\mathrm{std}\left(\frac{Z-\bar{Z}}{\sigma Z}\right)}$$

where *Z* represents the centroid displacement in the sampling sequence, and $\overset{-}{Z}$ denotes the mean value, yielding a zero-centered deviation measurement. The standard deviation σZ is incorporated in the denominator to normalize the data, eliminating dimensional effects and enabling cross-experimental comparison of fluctuation patterns.

The parameter optimization described above provides the foot and CoM trajectories, ensuring that the gait of the robot satisfies the ZMP stability constraints. However, this trajectory cannot be directly applied to the motion control of the robot. There are two primary reasons for this. Firstly, the aforementioned motion fails to ensure that the generated step size and height align with the mechanical structural parameters of the robot. Second, the previously mentioned optimization results do not guarantee proper coordination between the stride and body of the robot.

In the process of human walking, there is a good linear relationship between step size and height. To ensure that the step size of the robot walking conforms to the mechanical dimensions of the robot and improves the appearance coordination of the robot walking, this paper introduces a second fitness function, as shown in Equation (16).

(16) $$fitness_{\mathrm{atti}}=\frac{0.54}{k_{c}F_{x}-\left(H+c_{x}\right)+1.321}$$

Among these variables, $k_{c}$ represents the adjustment coefficient, $F_{x}$ denotes the step size of the movement along the *x*-axis of the robot, *H* corresponds to the height of the robot in the gravitational direction, and $c_{x}$ is a constant. Furthermore, the sum of *H* and $c_{x}$ must fall within the range of [1.64, 1.86].

This paper integrates the multidimensional objectives of stability and coordination into a single fitness function via weighted summation (Equation (17)). Unlike multi-objective approaches, this single-objective optimization method eliminates the need for Pareto front analysis or non-dominated sorting, thereby significantly improving computational efficiency.

(17) $$fitness=w_{1}fitness_{\mathrm{ZMP}}+w_{2}fitness_{\mathrm{atti}}+w_{3}fitness_{\mathrm{dev}}-10\times V_{p}$$

where $w_{1}$, $w_{2}$, and $w_{3}$ are positive weighting coefficients. Since the stability of the robot’s walking takes higher priority over coordination, in this paper, $w_{1}$ is set to 0.52, $w_{2}$ to 0.28, and $w_{3}$ to 0.20. $V_{p}$ is the penalty term; it equals 1 when the centroid height falls below 0.25 m, and 0 otherwise.

### 3.3. Research on Vestibular Reflex in Sloping Terrain

**Figure 7:**
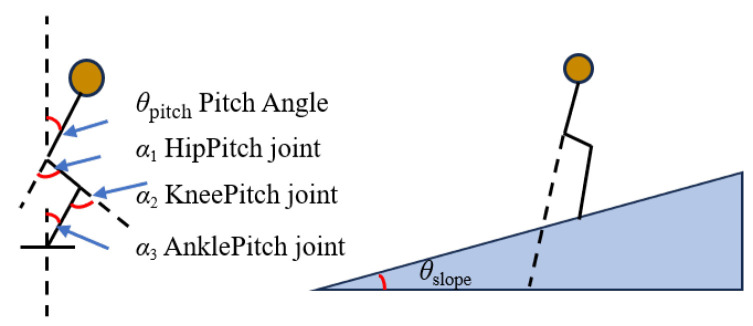
Human joint angle and slope angle.

When the robot directly interacts with the environment, it must sense external feedback and adjust its behavior to maintain stability. When humans move on inclined terrain, they rely on the vestibular reflex to adjust their posture and maintain balance. Drawing on the biological mechanism of the human vestibular reflex, this paper simulates the inner ear vestibule through trunk inertial sensors to detect changes in robot posture. As illustrated in Figure 7, the terrain slope angle can be derived from the humanoid robot’s attitude and joint angle measurements.

The method for calculating the slope angle $\theta_{\mathrm{slope}}$ is given by Equation (18).

(18) $$\theta_{\mathrm{slope}}=\alpha_{1}+\alpha_{3}-\alpha_{2}-\theta_{\mathrm{pitch}}$$

To quantify the consistency between the response of the robot and its motion intentions, this paper establishes a reflex mechanism for the robot using Tegotae theory and couples the environmental perception information with the CPG oscillator. First, based on the slope angle $\theta_{\mathrm{slope}}$ and the adjustment amount $comp_{x}$ of the CoM displacement in the *x*-direction, a *T*-function is created to quantify Tegotae, as shown in Equation (19).

(19) $$T\left(comp_{x},\theta_{\mathrm{slope}}\right)=C\left(comp_{x}\right)S\left(\theta_{\mathrm{slope}}\right)=\left(thre-\sin\left(k_{c}\left(comp_{x}-thre\right)\right)\right)\theta_{\mathrm{slope}}$$

where $comp_{x}$ is the feedback control variable, thre is the adjustment threshold, and $k_{c}$ is the gain constant. $C\left(comp_{x}\right)$ represents the intention of the controller, and $S\left(\theta_{\mathrm{slope}}\right)$ indicates the response obtained from the environment. During an uphill motion, the CoM needs to lean forward to avoid tipping backward. During a downhill motion, the CoM needs to adjust backward to prevent tipping forward. When the *T*-function value is large, it indicates that the slope of the terrain is steep, and the CoM adjustment amount is close to the threshold thre, meaning a significant correction to the CoM position is required. Based on this, a local perception feedback f can be designed to represent the increment of the *T*-function value by adjusting compx. For continuous systems, *f* is expressed as the partial derivative of the *T*-function with respect to the control variable, as shown in Equation (20).

(20) $$f=k_{T}\frac{\partial T}{\partial comp_{x}}=-k_{T}k_{c}\cos\left(k_{c}\left(comp_{x}-thre\right)\right)\theta_{\mathrm{slope}}$$

where $k_{T}$ is the gain constant. When the adjustment amount exceeds the threshold, the adjustment is considered too large, and the *T*-function expresses that the current behavior is undesirable. Therefore, it needs to be corrected with a negative value.

The feedback term of the CoM layer in the Kimura CPG is designed as shown in Equation (21).

(21) $$feed_{com}^{\left\{e,f\right\}}=\left\{\begin{array}{cc} -k_{T}k_{c}\cos\left(k_{c}\left(comp_{x}-thre\right)\right)\theta_{\mathrm{slope}}, & \left|\theta_{\mathrm{pitch}}\right|\in \left(\theta_{m},\theta_{thre}\right)\\ 0, & \left|\theta_{\mathrm{pitch}}\right|\in \left(0,\theta_{m}\right) \end{array}\right.$$

Here, due to the slight fluctuations in the attitude angle during the walking process of the robot, it is necessary to design the minimum deviation angle $\theta_{m}$. $\theta_{thre}$ is the critical attitude angle at which the robot transitions between stable and overturned states, which can be measured by attitude sensors.

## 4. Simulation and Experiment of Humanoid Robot Walking

### 4.1. CPG Parameter Optimization and Trajectory Generation

**Figure 8:**
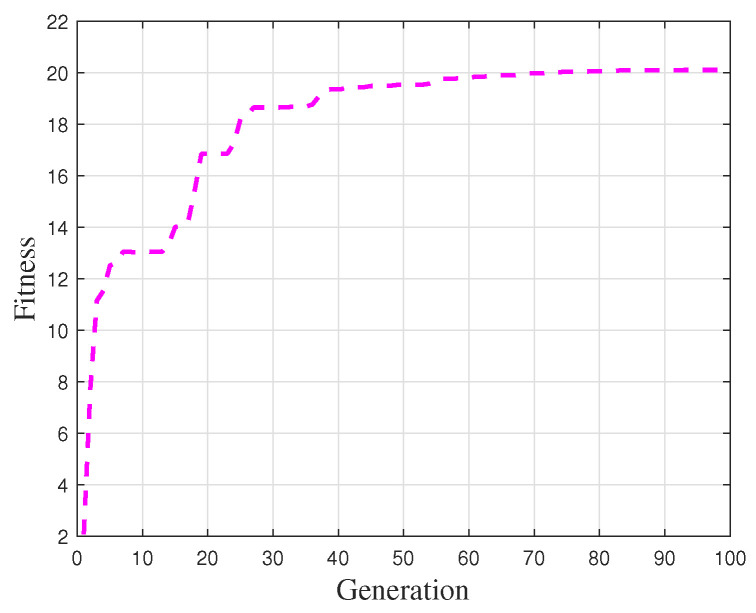
Fitness function values. Converge to the optimal level within 100 generations.

For the parameter settings of the genetic algorithm optimization process, the population size was set to 100, the number of generations to 100, the crossover probability to 0.8, and the mutation rate to 0.29. Based on these settings, the gait constraints of the robot were optimized. The fitness function value progression is illustrated in Figure 8.

As shown in the Figure 8, the optimization of the robot trajectory parameters based on the genetic algorithm yields good results. The fitness function value increases significantly from generations 1 to 30, and eventually converges at 20.106. The objective function, being the reciprocal of the fitness function, has a value of 0.0497. The objective function designed in this paper describes the distance between the ZMP landing point and the stability region center, as well as the deviation of the gait shape from the ideal state.

Reference [20] introduced an improved BPSO algorithm with a stability optimization objective centered on a survival-time deviation term (Equation (22)). The objective function incorporates the lateral offset distance ($cost_{\mathrm{dev}}$), forward distance deviation ($cost_{\mathrm{fd}}$), and survival time deviation ($cost_{\mathrm{ld}}$).

(22) $$Pe=w_{1}cost_{\mathrm{dev}}+w_{2}cost_{\mathrm{fd}}+w_{3}cost_{\mathrm{ld}}-50\times V_{p}$$

where the Pe is the penalty function. However, this approach requires nearly 1000 iterations to achieve optimal performance. In contrast, the ZMP-based optimization scheme proposed in this study converges to a near-optimal solution within 100 generations, significantly improving computational efficiency while maintaining comparable performance. This advantage primarily stems from two factors. First, metrics such as survival time are posterior statistics that lack explicit physical correlation with the dynamic parameters of the gait generator. In comparison, ZMP serves as a direct physical indicator that enables continuous and differentiable fitness evaluation with richer gradient information, thereby accelerating convergence. Second, the proposed method does not rely on iterative kinematic model computations, further simplifying the optimization process and enhancing computational efficiency.

The optimization results for the internal parameters of the CoM CPG, as well as the coefficients of the mapping function, are presented in Table 1.

The optimization results for the internal parameters and the mapping function coefficients of the foot trajectory CPG are presented in Table 2.

**Figure 9:**
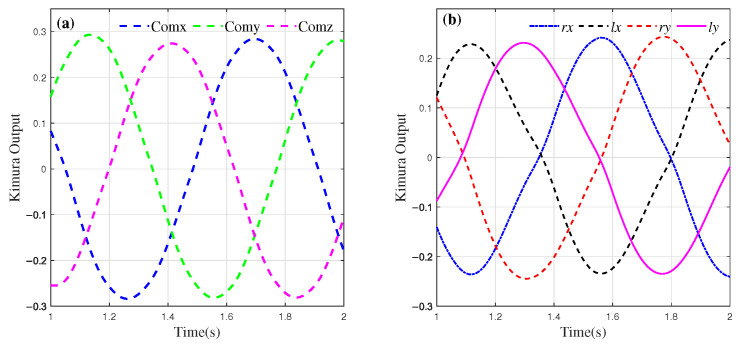
Kimura CPG output after optimization. (**a**) CoM CPG output. (**b**) Foot CPG output. The components of the four dimensions maintain a certain phase difference.

Following optimization using the genetic algorithm, the output signals of the CoM trajectory planner and the foot-end trajectory planner are presented in Figure 9a and Figure 9b, respectively.

**Figure 10:**
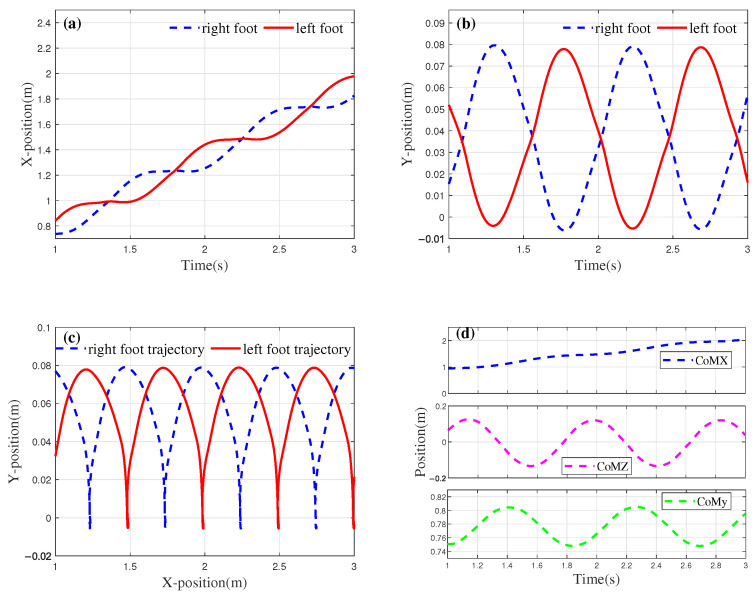
Schematic diagram of motion path of CoM. (**a**) The generated $foot_{x}$ trajectory. (**b**) The generated $foot_{y}$ trajectory. (**c**) Foot trajectory in the *x*-*y* plane. (**d**) Schematic diagram of motion path of CoM.

The foot trajectory generated based on the optimization results is shown in Figure 10. Figure 10a illustrates the displacement variations of the left and right feet in the *x*-direction over each unit of time. From the figure, it can be seen that after the biped’s displacement increases in the first half of the cycle, the displacement in the *x*-direction remains almost constant during the second half of the cycle, indicating that the foot is in the stance phase. Additionally, the distance between the two contact points represents the step length of the robot, with $F_{x}$ equal to 0.27 m.

In the design of the fitness function, a linear relationship between height and step length is introduced. Based on the physical parameters of the robot prototype, the height *H* is set to 1.21 m, and $k_{c}$ is set to 1.78. Substituting *H* into Equation (16) yields an ideal step length of 0.33 m. The optimization result has an error of 0.06 m, corresponding to a relative error rate of 18.2%. During the parameter optimization process, the weight $w_{2}$ of the step length coordination index was set to 0.28, which sacrifices some coordination to achieve better gait stability. Therefore, the error primarily arises from the weight $w_{2}$ in the fitness function. Figure 10b shows the motion of both feet in the *y*-direction (gravity direction), where the peaks of the rhythmic signal represent the step height. Figure 10c displays the shape of the gait in the *x*-*y* plane. Since the connection weight was not adjusted during the parameter optimization process, the phase difference between the two feet in both the *x* and *y* dimensions remains unchanged. The results presented in Figure 10 demonstrate that the CPG planner developed in this study is capable of generating gaits that adhere to the biomechanical principles governing human foot swing and ground contact. The optimized CoM trajectory of the robot is shown in Figure 10d.

In designing the CoM trajectory, this study establishes a guideline ensuring that the oscillation of the CoM in the *z*-direction aligns with the motion of the right foot. Specifically, when the right foot makes contact with the ground, the CoM is expected to shift to the right. In other words, the offset value in the *z*-direction, as illustrated in Figure 10d, should correspond to the trough of the cycle at that moment. The trajectory of the CoM in the *x*-direction aligns well with the foot trajectory. In the *z*-direction, the oscillation amplitude does not exceed the boundary values of the feet coordinates during the standing phase, with an oscillation amplitude of 0.266 m. Additionally, the maximum displacement of the CoM in the *y*-direction is 0.042 m, with the oscillation in the *y*-direction being influenced by the height of the robot and foot movement.

**Figure 11:**
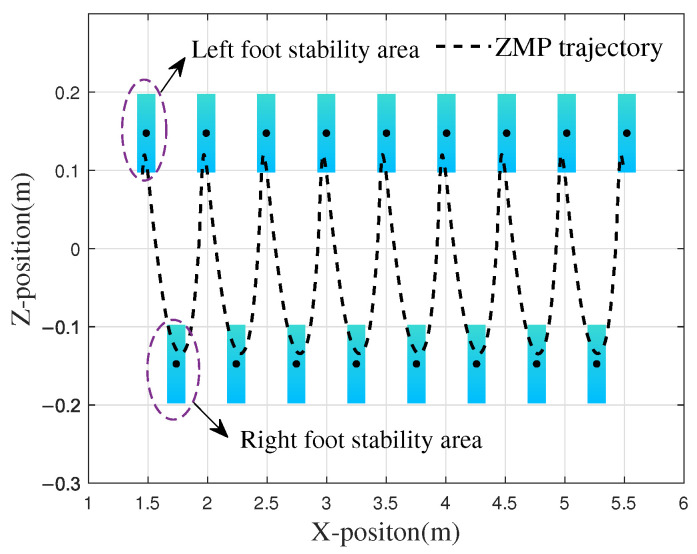
ZMP locus and landing point distribution. Walk in the direction of increasing *x*.

Based on the known foot and CoM trajectories, the ZMP trajectory in the *x*-*z* plane can be calculated using Equation (11). The rectangular stability region and the ZMP trajectory, with the ankle joint of the biped as the center, are shown in Figure 11.

The figure depicts nine gait cycles during the walking process of the robot, with the ZMP consistently remaining within the envelope polygon of the sole during single-foot support. When the right foot touches the ground at 5.48 m, the maximum distance between the ZMP and the center of the stable region is 0.0485 m, while the stability margin ($D_{s}$) at this moment is 0.021 m. During the walking process, the robot also engages in bipedal support. As shown in Figure 11, the variation range of the ZMP always remains within the closed polygon defined by the boundaries of the bipedal support region. The optimization results obtained through the genetic algorithm demonstrate that the planned robot gait achieves excellent stability.

### 4.2. Simulation Verification of Walking on Flat Ground and Step Size Optimization

**Figure 12:**
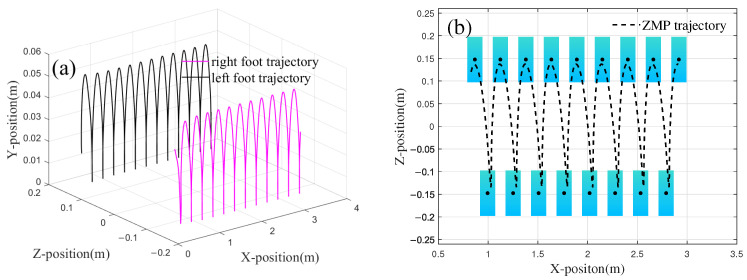
Robot comparison trajectory 1. (**a**) Robot foot trajectory. (**b**) ZMP locus and landing point distribution.

In the previous section, gait stability was validated by calculating the ZMP and foot contact point coordinates. This section focuses on verifying the optimization effectiveness of the gait using $fitness_{atti}$. The primary objective of $fitness_{atti}$ is to constrain the step length to ensure that the optimized gait remains within the motion range while simultaneously enhancing the coordination of the gait. In this study, two different gaits are generated without applying $fitness_{atti}$, and their performance is compared with the gait presented in Section 4.1. The comparison is conducted in the Adams simulation environment using a virtual prototype. The first generated gait without $fitness_{atti}$ is shown in Figure 12.

**Figure 13:**
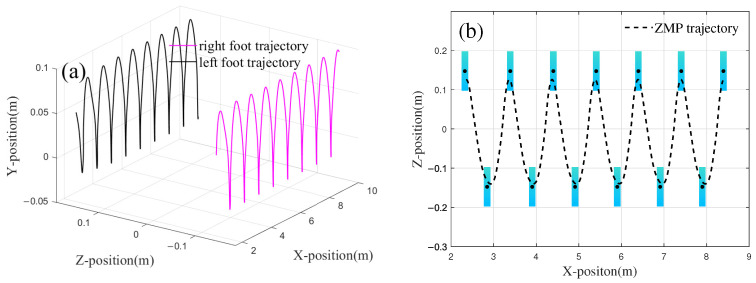
Robot gait comparison trajectory 2. (**a**) Robot foot trajectory. (**b**) ZMP locus and landing point distribution.

In this gait, the step length is 0.107 m, and the step height is 0.048 m. The relationship between the ZMP and foot contact point distribution is shown in Figure 12b. From the figure, it can be observed that the ZMP of this trajectory is positioned closer to the center of the single-foot contact area, and when the *x*-coordinate reaches 2.81 m, the ZMP approaches the boundary of the polygon. At this point, the stability margin $D_{s}$ is 0.05 m, indicating that this gait is more stable. The second gait generated is shown in Figure 13a. In this gait, the step length is 0.53 m, the stride length is 1.06 m, and the step height is 0.096 m. The ZMP and the double-foot trajectory are shown in Figure 13b. The ZMP trajectory indicates that the gait of the robot in this case also maintains a high level of stability.

Since the designed trajectory primarily targets the foot, without imposing constraints on joint movements and the amplitude of the CPG itself, it is easy to encounter significant differences in the step lengths of the two generated gaits. Although both gaits satisfy the ZMP stability constraint used in this paper, it cannot be assumed that a stable gait is necessarily a reasonable gait. To verify the feasibility of the obtained trajectories, a virtual lower limb model of the robot is established for gait simulation.

**Figure 14:**
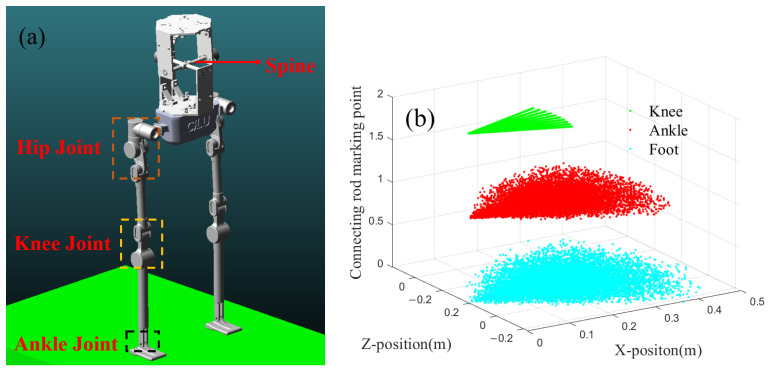
Schematic diagram of robot prototype. (**a**) Virtual prototype of robot. (**b**) Robot linkage motion range.

The virtual prototype of the humanoid robot is shown in Figure 14a. The lower limb has a height of 0.71 m, with three degrees of freedom at the hip joint and one degree of freedom each at the knee and ankle. A mass block simulates the torso, located at the midpoint between the two hips, which is considered the CoM. Figure 14b illustrates the range of motion in the sagittal plane across three levels, with the hip joint serving as the origin and the knee joint, ankle joint, and toe joint representing the endpoints of the connecting rods.

The blue scatter plot in Figure 14b illustrates the range of motion of the toe. As observed from the graph, the maximum step length of a single leg is constrained to less than 0.5 m. The range of motion depicted is influenced by the length of each connecting rod, with the knee joint exhibiting the smallest range of rotation in the sagittal plane. Compared to the lower limbs, the foot has a shorter length, resulting in a similar range of motion for both the foot and the ankle joint.

**Figure 15:**
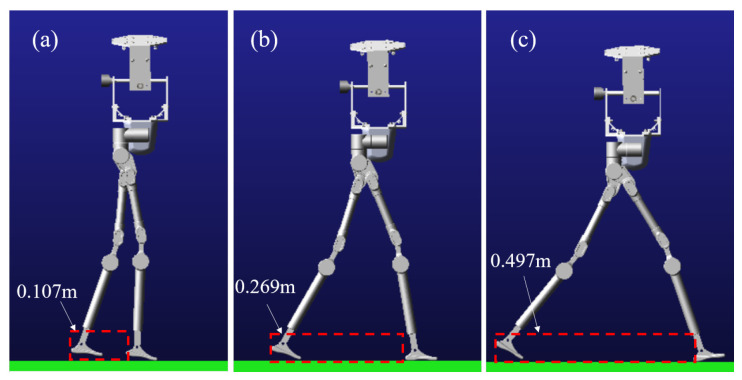
Step length diagram of robot virtual prototype: (**a**) comparison trajectory 1, (**b**) near natural trajectory with $fitness_{atti}$, (**c**) maximum step size under mechanism limit.

Based on the obtained foot and CoM trajectories, the inverse kinematics model of the robot must be used to calculate the joint motion trajectories for the lower limbs and drive the robot in the simulation environment. Among the trajectories, the second set has a larger stride length, and the range of joint motion exceeds the reachable space of the robot’s joints, making it an invalid gait. In the virtual simulation environment, the gaits from Section 4.1 and the comparative trajectory (Trajectory 1) are used to simulate the robot. The two sets of trajectories in the simulation environment and the maximum step size are presented in Figure 15.

In this figure, case (a) demonstrates that without $fitness_{atti}$ optimization, the robot achieves only a 0.107 m step length. This suboptimal gait pattern produces uncoordinated locomotion due to insufficient stride dimensions. By contrast, $fitness_{atti}$-optimized parameters yield a 0.269 m step length (case (b)), where the gait characteristics properly match the robot’s physical proportions. The resulting walking pattern exhibits significantly improved coordination and natural appearance. Furthermore, case (c) reveals the robot’s maximum achievable step length of 0.497 m. These comparative results conclusively validate the essential role of $fitness_{atti}$ optimization in generating biomechanically coordinated gaits.

**Figure 16:**
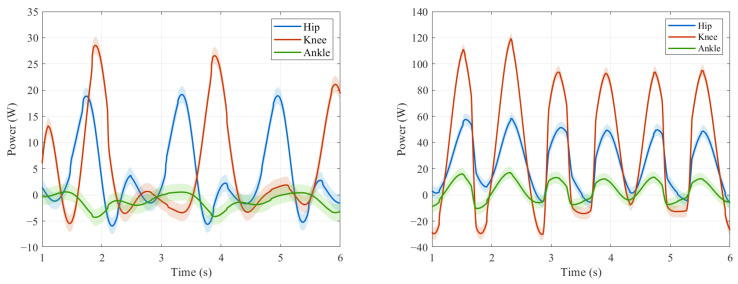
Comparison of joint energy consumption. (**a**) joint power consumption of near natural gait. (**b**) Joint power consumption of uncoordinated gait (comparison trajectory 1).

The human body spontaneously adopts the most energy-efficient movement mode during walking, which indicates an inherent correlation between natural gait and minimizing energy consumption. For humanoid robots, optimizing gait coordination is essentially equivalent to minimizing power consumption. When the step size is in the optimal biomechanical range, the peak power distribution of each joint in the lower limbs should exhibit dynamic equilibrium characteristics. Figure 16 compares the lower limb joint power distribution characteristics of two typical step size patterns (0.269 m near natural gait and 0.107 m uncoordinated gait) in Figure 15.

This paper uses the dimensionless cost of transport (CoT) as the primary metric for evaluating the energy efficiency of humanoid robots, as shown in Equation (23).

(23) $$\mathrm{CoT}=\frac{E}{mgd}=\frac{P}{mgv}$$

where *P* represents the average power, *D* is the displacement, and *V* is the average speed. The total energy consumption *E* is obtained by integrating the instantaneous power of each joint driver over time, as described in Equation (24).

(24) $$E=\int_{t_{1}}^{t_{2}}P\left(t\right)dt$$

Currently, commonly used CPG optimization schemes, such as references [20,34], mainly focus on the walking stability of the optimization objective. This approach often overlooks key aspects such as naturalness of movement and energy consumption. The contribution of gait coordination and motion naturalness to peak power suppression and energy redistribution can be quantitatively evaluated using the system-level energy consumption metric. The corresponding experimental data are presented in Table 3. Under the experimental condition of keeping the same distance and time, the CoT of near natural gait was 1.75, while that of uncoordinated gait was 4.08. Compared with the optimization scheme that only considers stability, the energy consumption has been reduced by 57.1%. The simulation results show that the coordinated gait mode significantly reduces the energy consumption of the system, and its energy efficiency characteristics conform to the energy optimization law in human biomechanics.

### 4.3. Changing Slope-Terrain Adaptive Walking

**Figure 17:**
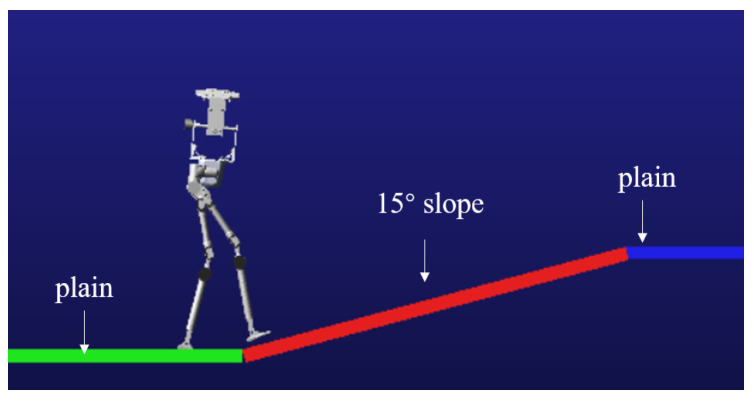
Robot virtual prototype and slope environment. In the schematic, green and blue areas represent flat ground, while red indicates a sloped surface.

To verify the stability of the robot while walking on a slope with varying gradients, a simulation test is conducted. In the Tegotae function, the parameters $k_{T}$ is assigned a value of −0.01, $k_{c}$ is set to 5.2, and the threshold thre is defined as 0.18. In the simulation environment, the slope angle is configured to 15 degrees. The robot and the walking environment are shown in Figure 17.

**Figure 18:**
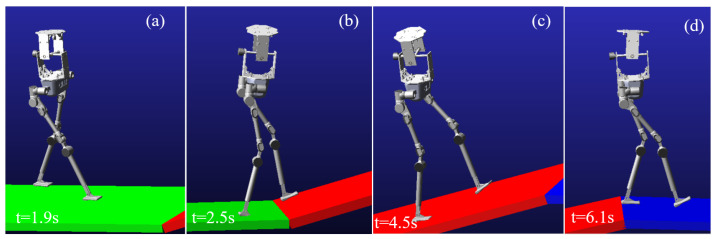
Walking simulation snapshot. (**a**) Initiate straight-legged walking on flat ground. (**b**) Transition to a bent-knee posture and initiate leg lifting. (**c**) Adapt to slope walking by shifting the center of gravity forward. (**d**) Conclude the slope traversal and return to walking on flat terrain.

During the simulation, the robot begins walking from a flat surface, reaches the slope at 2.5 s, and transitions back to the flat surface at 6.1 s. The walking process of the robot in the virtual environment is shown in Figure 18.

**Figure 19:**
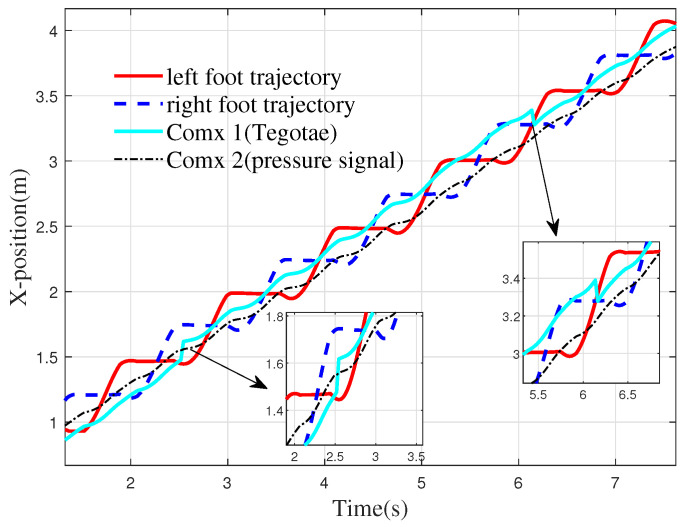
Robot foot and CoM movement. Comx 1 is the CoM trajectory with Tegotate feedback control used in this paper, and Comx 2 is the CoM trajectory with plantar pressure feedback control designed in reference [34].

From the simulation process, it can be observed that the robot does not experience tipping during the walking process. However, the sliding of the foot occurs when walking on the slope. The sliding phenomenon is primarily related to the physical parameter settings in the simulation environment. The motion trajectory of the robot in the *x*-direction is shown in Figure 19.

From Figure 19, it can be seen that when the robot walks on flat ground, the Comx 1 is close to the lower edge of the foot trajectory. In this state, when the robot transitions onto the slope, it is prone to tipping backward. In this paper, the Tegotae function simulates the human vestibular reflex and adjusts the posture of the robot. When the robot reaches the slope at 2.52 s, the CoM is adjusted forward by 0.09 m to improve walking stability. When the robot transitions from the slope to flat ground at 6.1 s, the slope angle decreases. To enhance stability, the CoM needs to shift backward. According to the Tegotae function, the robot adjusts its CoM backward by 0.10 m when walking back onto the flat surface.

Reference [34] proposed a feedback strategy based on plantar pressure, formulated as follows:

(25) $$\left\{\begin{array}{c} feedback1=FsrR-FsrL\\ feedback2=-feedback1 \end{array}\right.$$

where $feedback_{1}$ and $feedback_{2}$ denote feedback terms for a pair of antagonistic neurons, and FsrR and FsrL represent the total pressure signals measured by the right and left foot plantar sensors, respectively. This method judges the robot’s balance by checking whether the center of gravity lies within the stability region of the Center of Pressure (COP) via the difference in foot pressures. As observed in the Comx 2 trajectory in Figure 19, under this feedback mechanism, the robot exhibits no significant forward adjustment of the CoM after slope ascent. Moreover, the x-component of the CoM trajectory remains closer to the bipedal support boundary. The projection of the center of mass in the x-direction remained within the COP region for only 78.9% of the time in the baseline method. In contrast, the Tegotae-based approach achieved a markedly higher retention rate of 88.3%, representing an improvement of 9.4 percentage points. These results underscore the enhanced robustness of the proposed method.

**Figure 20:**
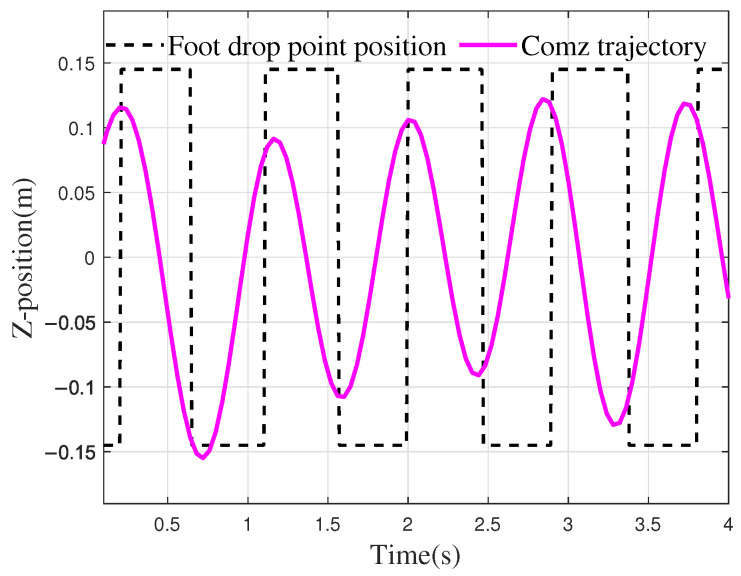
Trajectoryof robot foot and CoM.

**Figure 21:**
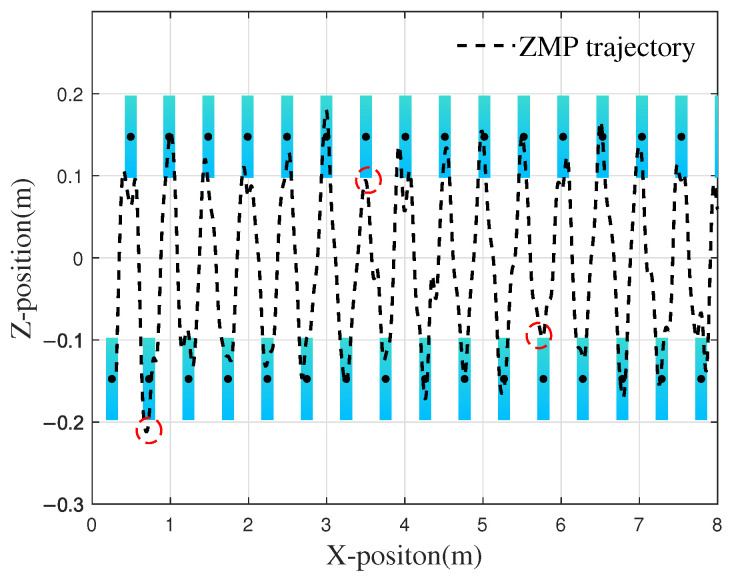
ZMP distribution of robot walking on the slope.

From Figure 20, it can be seen that during the walking process in the simulation environment, the CoM oscillation in the *z*-direction shows a trend consistent with the foot landing. For example, at 1.5 s, when the robot is in the right foot support phase of the walking cycle, the CoM is adjusted approximately 0.11 m to the right to maintain balance. After 2.3 s, there is a phase difference between the CoM oscillation and the single support phase, which is mainly due to the fact that the trajectory planning for both the CoM and the foot endpoint did not fully align in frequency and period. However, since the time difference between the lateral adjustment of the CoM and foot landing is relatively short, the robot does not experience tipping. To evaluate the influence of phase deviation between the CoM and foot trajectory on stability, the stability margin was computed. At 3.30 s, which corresponded to a maximum time deviation of 0.10 s, the stability margin was measured at 0.034 m. This confirms that the ZMP remained well within the support polygon without stability being compromised. Further analysis under high-dynamic conditions (Comparison Trajectory 1, higher speed) showed a time deviation of 0.097 s and a stability margin of 0.031 m. The ability to maintain a similar stability margin across different gait modes demonstrates the robustness of the control architecture. The ZMP distribution during the walking simulation process of the robot is shown in Figure 21.

As illustrated in Figure 21, the distribution of the ZMP during locomotion predominantly remains within the support area defined by both feet. On level ground, when the robot progresses to point A at a distance of 0.70 m, an excessive adjustment of the CoM along the z-axis causes the ZMP to slightly exceed the support area, with a deviation of 0.01 m, increasing the risk of instability. When the right foot lands at point B, the ZMP fails to consistently remain within the support polygon, with the minimum distance to the stability boundary being 0.005 m. At point C, the right foot makes contact with the ground during the transition between the supporting legs, and the ZMP falls at the boundary of the stable region. In these three scenarios, the potential for the robot to tip over arises; however, as this study defines the single-legged stability domain as the rectangular envelope surface centered on the contact area, the deviations beyond the boundary remain minor, enabling the robot to maintain stable locomotion. Furthermore, from 1 s to 7 s, the average deviation ($D_{s}$) per gait cycle is observed to be 0.02 m. Except during phases of single-foot support and foot transition, the ZMP distribution consistently resides within the enveloped region defined by both feet, thereby ensuring stability during walking.

### 4.4. Slope Walking Experiment of Humanoid Robot

**Figure 22:**
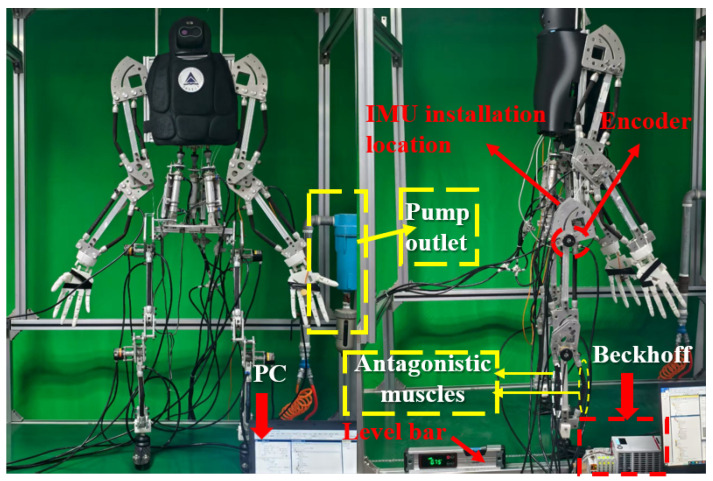
Prototype of pneumatic muscle humanoid robot.

This study is based on a self-developed pneumatic muscle humanoid robot to conduct walking performance tests. The physical prototype is shown in Figure 22.

Based on the motion trajectory defined in the previous section, a system dynamics model is established, and the three-element constitutive model is employed to calculate the optimal control pressure for each pneumatic muscle. For the specific modeling approach, refer to Reference [36].

**Figure 23:**
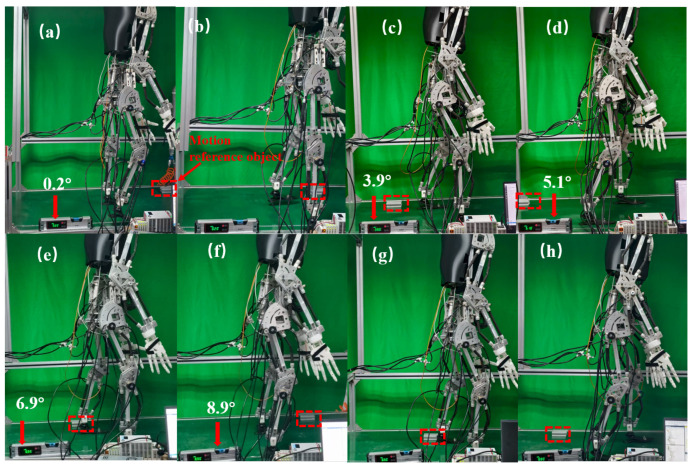
A snapshot of humanoid robot walking on flat ground and slopes. (**a**,**b**) stage 1: Walking on flat ground. (**c**–**h**) stage 2: Walking on a variably sloped surface, with the inclination increasing from 0° to a maximum of 8.9°. The subfigures depict representative walking snapshots at slopes of 3.9°, 5.1° and 6.9°.

To verify the effectiveness of the CPG walking control strategy, this study constructed an experimental environment with an integrated adjustable slope conveyor belt and installed a high-precision IMU sensor at the center of the robot’s two hip joints. The control performance was evaluated by collecting real-time attitude angle data. Figure 23 shows an experimental snapshot of the robot walking on a gradient-adjustable conveyor belt.

Figure 23a,b illustrate the first stage of walking, during which the robot moves on a nearly horizontal plane with a 0.2° inclination. As shown in panel (a), the robot lifts its right leg at 0.6 s; panel (b) depicts the left leg being lifted at 1.2 s. Figure 23c–h present the second stage of walking under variable slope conditions. Due to the physical constraints of the experimental setup, the maximum slope achievable was limited to 8.9°. At this inclination, the conveyor belt facilitates the robot’s transition from level-ground walking to slope walking. Panel (c) captures the robot’s walking posture as the slope gradually increases to 2.9°. At this point, the gait pattern shifts from straight-knee walking to bent-knee walking. Panels (d) and (e) represent the robot’s walking postures at slopes of 5.1° and 6.9°, respectively. Panels (f) to (h) display a complete gait cycle at the maximum slope of 8.9°. The robot completed postural adjustment for inclined standing at 15.9 s, characterized by flexed lower limbs, an upright stance, and a forward-leaning upper body to adjust the center of gravity. Subsequently, as shown in panel (g), the robot steps forward with the right leg at 17.2 s, followed by the left leg stepping forward at 18.4 s in panel (h).

**Figure 24:**
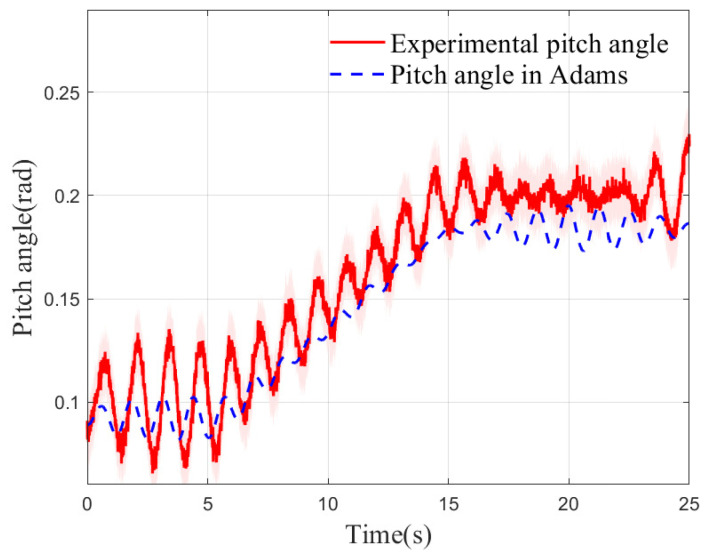
The body attitude in pitch planes.

The robot was driven from a planar surface onto a gently sloped incline, with its posture and pitch angle variations depicted in Figure 24. At 5.2 s, the inclination angle of the conveyor belt was gradually increased from an initial value near 0° to a final slope of 8.9°. As the slope increased, the pitch angle of the robot in the forward direction increased synchronously. When the slope stabilized at the maximum inclination of 8.9° at 14.9 s, the amplitude of the pitch angle also converged, eventually maintaining fluctuations around approximately 0.2 rad. Due to the gap from the simulation to the experiment, although there are differences in the steady-state amplitude, the time history curve of the pitch angle measured in the experiment is in good agreement with the Adams simulation results in the main characteristics. The experimental results indicate that the Tegotae mechanism proposed in this study can be effectively integrated into the CPG-based feedback control system. By sensing slope changes in real-time and dynamically adjusting the spatial position of the center of mass, the mechanism significantly enhances the robot’s disturbance rejection and balance maintenance capabilities. This control strategy enables the humanoid robot to sustain dynamic stability on varying slopes and achieve continuous, smooth autonomous locomotion.

## 5. Conclusions

This study proposes and validates a biomimetic motion control framework that integrates a CPG with a vestibular reflex mechanism to enhance humanoid robot locomotion in unstructured terrains. First, a CPG planner, designed based on human gait characteristics, effectively generates smooth and natural motion patterns in Cartesian space. To mitigate the mismatch between the mechanical structure and the generated gait, this study developed a gait with high dynamic stability by incorporating body posture constraints into a multi-objective optimization framework. Simulation results demonstrate that the optimized gait achieves a balance between motion performance and energy efficiency. Second, a reflex control strategy inspired by the Tegotae mechanism enables real-time adaptation to slope variations. Simulations verify the feasibility of the proposed framework in achieving stable walking on a 15° slope, while physical experiments demonstrate that the robot can stably transition from level ground to an 8.9° slope, significantly improving robustness on inclined surfaces. Collectively, these findings suggest that the proposed biomimetic control strategy provides an effective solution for enhancing humanoid robot locomotion in complex environments. Although constrained by site conditions (such as the maximum available slope angle), the results strongly support the potential of this framework.

Although this study has demonstrated the robot’s ability to stabilize locomotion on slopes up to 8.9°, certain limitations remain. The trajectory generation method proposed herein processes centroid and foot trajectories separately without integrating them into a unified CPG network, which restricts synchronization between control levels. Future research will focus on developing heterogeneous multi-layer CPG networks to better emulate human motion coordination mechanisms, thereby more fully utilizing the innate synchronization capabilities of CPG. Another key direction will involve advancing CPG from an offline planning tool to an online adaptive controller capable of responding to more complex and dynamic environments. Furthermore, investigating stable locomotion across a broader spectrum of complex terrains—such as stairways and unstructured outdoor paths—as well as under external disturbances will constitute a major emphasis in subsequent research.

## Figures and Tables

**Table 1:** Results of CoM trajectory parameter optimization.

Parameter	Value
$T_{r}$, $T_{a}$	0.123, 0.255
$s_{0}$, β, *a*	0.365, −3.614, −1.930
$\eta_{x}$, $\eta_{y}$, $\eta_{z}$	0.338, 0.776, −0.0072
$\varsigma_{x}$, $\varsigma_{y}$, $\varsigma_{z}$, $\lambda_{c}$	0.197, 0.101, 0.451, 0.586

**Table 2:** Results of foot trajectory parameter optimization.

Parameter	Value
$T_{r}$, $T_{a}$	0.139, 0.246
$s_{0}$, β, *a*	0.375, −3.414, −1.790
$x_{0}$, $z_{0}$	0.238, 0.037
$C_{x}$, $C_{y}$, $\delta_{f}$	0.388, 0.176, 0.555

**Table 3:** Results of gait energy efficiency comparison.

Joint	Consumption Index	Near Natural Gait	Uncoordinated Gait
Hip	Total Energy (J)	44.9	247
Knee	Total Energy (J)	90.7	425.3
Ankle	Total Energy (J)	4.4	54.3
Total	CoT	1.75	4.08

## Data Availability

The original contributions presented in this study are included in the article.

## Nomenclature

The abbreviations and symbols used in the manuscript are shown in the naming cross-reference table below:

symbols	Physical meaning	abbreviations	Full name
$foot_{x}$	Foot trajectory, *x*-component	CPG	Central Pattern Generator
$foot_{y}$	Foot trajectory, *y*-component	ISB	International Society of Biomechanics
${\mathrm{CoM}}_{x}$	CoM trajectory, *x*-component	ZMP	Zero Moment Point
${\mathrm{CoM}}_{y}$	CoM trajectory, *y*-component	CoM	Center of Mass
${\mathrm{CoM}}_{z}$	CoM trajectory, *z*-component	GA	Genetic Algorithm
$D_{s}$	Stability margin	CoT	Cost of Transport
fitness	Fitness function	/	/
$\theta_{\mathrm{pitch}}$	Torso pitch angle	/	/
$\theta_{\mathrm{slope}}$	Slope angle	/	/
